# The association between hip morphology parameters and nineteen-year risk of end-stage osteoarthritis of the hip: A nested case–control study

**DOI:** 10.1002/art.30523

**Published:** 2011-11

**Authors:** Alex S Nicholls, Amit Kiran, Thomas C B Pollard, Deborah J Hart, Charlotte P A Arden, Tim Spector, H S Gill, David W Murray, Andrew J Carr, Nigel K Arden

**Affiliations:** 1 Nuffield Orthopaedic Centre and University of OxfordOxford, UK; 2 St. Thomas' Hospital and Kings CollegeLondon, UK; 3 Nuffield Orthopaedic Centre and University of Oxford, Oxford, UK, and University of SouthamptonSouthampton, UK

## Abstract

**Objective:**

Subtle deformities of the hip joint are implicated in the etiology of osteoarthritis (OA) of the hip. Parameters that quantify these deformities may aid understanding of these associations. We undertook this study to examine relationships between such parameters and the 19-year risk of total hip arthroplasty (THA) for end-stage OA.

**Methods:**

A new software program designed for measuring morphologic parameters around the hip was developed and validated in a reliability study. THA was the outcome measure for end-stage OA. A nested case–control study was used with individuals from a cohort of 1,003 women who were recruited at year 1 in 1989 and followed up to year 20 (the Chingford Study). All hips with THA by year 20 and 243 randomly selected control hips were studied. Pelvis radiographs obtained at year 2 were analyzed for variations in hip morphology. Measurements were compared between the THA case group and the control group.

**Results:**

Patients with THA had a higher prevalence of cam deformity than did their respective controls (median alpha angle 62.4° versus 45.8° [*P* = 0.001]; mean modified triangular index height 28.5 mm versus 26.9 mm [*P* = 0.001]) as well as a higher prevalence of acetabular dysplasia (mean lateral center edge angle 29.5° versus 34.3° [*P* = 0.001]; median extrusion index 0.25 versus 0.185 [*P* = 0.009]). Logistic regression analyses clustering by subject and adjusting for radiographic hip OA at year 2 showed that these morphologic parameters were still significantly associated with THA by year 20. The alpha angle and lateral center edge angle predicted the risk of THA independently when included in the same model.

**Conclusion:**

This investigation describes measurements that predict the risk of THA for end-stage OA by year 20, independently of the presence of radiographic hip OA at year 2. These measurements can be made on an anteroposterior pelvis radiograph, which is an inexpensive and commonly used clinical method of investigation.

Epidemiologic studies indicate that osteoarthritis (OA) of the hip frequently occurs in the absence of OA in other large joints, suggesting that local factors are important in its pathogenesis (1–5). Drawing on earlier reported theories (6, 7), Harris (8) suggested that subclinical biomechanical factors may be important in the development of hip OA. He rebuked theories that the majority of hip OA cases were “primary” or “idiopathic,” and he hypothesized that abnormal hip morphology predates onset of OA and is not secondary to the arthritis process (8). Several studies (9–13) have since provided evidence to support this hypothesis.

Specific abnormalities of hip morphology are now recognized as biomechanical risk factors for the development of OA of the hip (8–11, 14). The predominant mechanisms are acetabular dysplasia, whereby the shallow acetabulum results in focal loading of articular cartilage beyond its physiologic tolerance (10), and femoroacetabular impingement (3, 14), which occurs as a consequence of abnormal contact between the acetabular rim and femoral head–neck junction, resulting in damage to the chondral surface and labrum. Together with improved understanding of these deformities, parameters have been introduced to quantify them and enable classification of patients presenting with early hip disease (6, 15–19).

While associations of abnormal morphology and OA are established (13), we are lacking prospective longitudinal data that may provide more convincing evidence of a causal relationship, particularly in relation to femoroacetabular impingement. If subclinical deformities can be shown to be effective predictors of OA in the general population, it may be possible to identify hips that are at risk before they progress to end-stage OA, necessitating total hip arthroplasty. In addition, clinical trials of both surgical (20) and medical treatments for at-risk hips will mandate accurate morphologic classification.

Anteroposterior (AP) pelvis radiographs are the most commonly used imaging modality for hip joints because they are inexpensive, readily accessible, and available in large study cohorts (21–25). Not only are they the Osteoarthritis Research Society International–recommended gold standard for assessment of progression of hip OA (26), they also enable the recognition and quantification of deformities associated with dysplasia and femoroacetabular impingement (1, 8–11, 14–16, 23, 27).

The present study validated a new software model for rapidly quantifying morphologic parameters of the hip joint on an AP pelvis radiograph. This software was then used to analyze the AP pelvis radiographs of a group of healthy women, who were selected from the population using a random number generator. These same volunteers were then followed up for 19 years, and records of any total hip arthroplasty (THA) performed for end-stage OA were gathered. The aim of the study was to identify any relationship between morphologic parameters in the hip joint at baseline and risk of THA during the followup period.

## SUBJECTS AND METHODS

### Subjects

In 1989, all women ages 44–67 years who were registered at a London-based general practice were asked to take part in the Chingford Study (28, 29). A total of 1,003 subjects participated at year 1, with yearly clinic visits to year 10; morphometric, clinical, biologic, and radiographic measurements were obtained at these visits. Each woman had an AP pelvis radiograph taken at year 2. These radiographs were performed with the patient in the supine position and a small sand bag under the knees to minimize hip rotation. In subsequent years, clinic visits were less frequent; however, telephone questionnaires were continued annually through to the present day (year 20). These included details of any operations undergone in the previous year. Confirming that a patient had undergone THA for end-stage OA was done by contacting the patient's general practitioner and checking the medical records at the hospital at which the surgery was performed.

The present study selected all cases (hips) from the original cohort that had undergone THA between years 2 and 20 (40 hips in 31 individuals). An additional 114 individuals who had not undergone THA were selected as the control group using a random number generator. Thus, a total of 290 hips in 145 individuals were initially selected for study.

### Exclusions

Exclusion criteria were applied to ensure that year 2 radiographs were of a minimum acceptable standard of quality to be included in the present study. From the starting cohort of 1,003 individuals, 4 hip joints (2 individuals) were excluded due to poor radiograph quality. Poor radiograph quality was a subjective exclusion criterion applied by the principal investigator when a radiograph was either grossly over- or underexposed to the extent that constituent anatomic landmarks were not visible for the purposes of analysis. Three hip joints (2 individuals) were excluded because they already had a THA in situ. One hip joint was excluded because it had a dynamic hip screw in situ, indicating previous femoral neck fracture. Twenty-four hip joints (12 individuals) were excluded because they had excessive tilt, as measured according to the distance between the sacrococcygeal joint and the pubic symphysis (30, 31). A total of 32 hips in 17 individuals were excluded. Of the 32 excluded hips, 22 happened to be in the selected THA and control cohorts, leaving 25 hips that had undergone THA and 243 hips that had not.

### Radiographic assessment of morphology

Hip morphology was analyzed using a Matlab-based software package (Matlab R2009b; MathWorks) called Hip Morf 2.0. A prototype (Hip Morf 1.0) has been used in previous cohort studies at our institution (19, 32). The software allowed the user to click on each of the constituent anatomic landmarks needed to measure several morphologic parameters. Once all landmarks had been identified, the necessary angle, ratio, and linear measurements were automatically calculated. We selected a large group of commonly used morphologic parameters to be included in this process. Each radiograph was anonymized and read independently by 2 investigators.

### Reliability study

A formal reliability study was performed on anonymized AP pelvis radiographs. Intraobserver repeatability was assessed by 1 investigator reading 10 randomly selected blinded radiographs on 3 occasions. Interobserver reproducibility was assessed by 2 additional observers reading the same 10 radiographs. Morphologic measurements were compared using intraclass correlation coefficients for continuous data and the kappa statistic for the triangular index, which has a binary outcome.

### Statistical analysis

**Figure 1:**
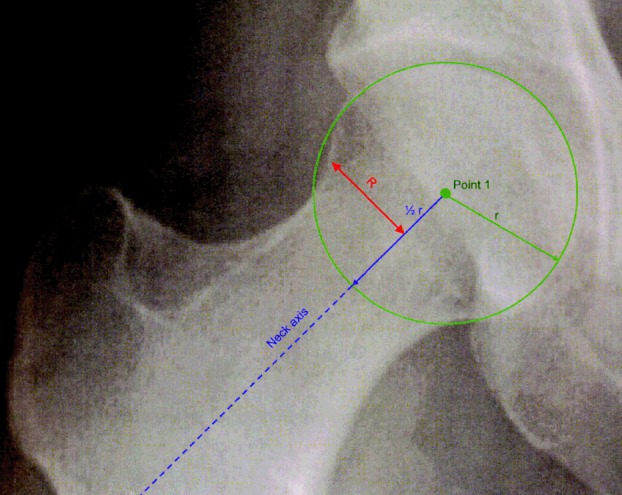
Illustration of the measurement of modified triangular index height (R; recorded in mm). In the original description of the triangular index (16), if R is greater than the radius (r) + 2 mm, a cam deformity is diagnosed. Point 1 = center of the femoral head.

For each morphologic measurement, the mean of the measurements from the 2 investigators was calculated to reduce variability in the data. This was justified because the reliability study demonstrated good concordance (see Results). Normally distributed variables were compared using the independent 2-tailed *t*-test, non-normally distributed variables were compared using the Wilcoxon rank sum test, and categorical data were compared using Fisher's exact test. Where positive associations were identified with THA, logistic regression analysis was performed using THA as a binary outcome. The model adjusted for baseline height, body mass index (BMI), age, joint space narrowing, and osteophyte grade. The triangular index (16) is reported as a binary outcome. Therefore, in order to enable this variable to be included in regression analyses, a modification was used whereby the actual distance R was recorded as the modified triangular index height (in mm) (see Figure 1). A long distance R implies a tendency to cam deformity.

Parameters that were statistically significant in the regression model were then combined in further multivariate analyses, using all of the same covariates, to assess whether they were independent predictors of THA. Logistic regression analyses were used with robust standard errors and clustering of hips by subject identifier code to account for the dependency of 2 hips from 1 subject. With 80% power at the 5% significance level, the present study was able to detect changes of 8.8% in the extrusion index ratio measurements, 2.72° in the acetabular index, 1.69 mm in the modified triangular index height (16), 12.02° in alpha angle, and 4.47° in lateral center edge angle.

Because both acetabular undercoverage (dysplasia) and overcoverage (pincer deformity) are implicated in the etiology of OA, kernel density plots were produced for the lateral center edge angle and acetabular index to evaluate the distribution of data. All statistical analysis was conducted using Stata SE, version 10 (StataCorp) and Matlab R2009b.

## RESULTS

The baseline clinical characteristics of the subjects included in this study (n = 135) were representative of the whole “Chingford 1,000 Women Study” (n = 1,003 subjects), as shown in Table 1. Patients were significantly older and taller at baseline than controls, as shown in Table 2. Patients also had a smaller joint space width than controls.

**Table 1:** Baseline characteristics of the subjects*

Characteristic	Full cohort (n = 1,003)	Subjects used in this analysis (n = 135)	Subjects not used in this analysis (n = 868)
Age, years	54 (49–60)	55 (50–60)	54 (49–59)
Height, mean ± SD meters	1.62 ± 0.059	1.61 ± 0.057	1.62 ± 0.060
Weight, kg	65 (58.5–73)	65.5 (59–71.8)	65 (58.4–73.4)
BMI, kg/m^2^	24.86 (22.63–27.61)	24.83 (22.88–27.32)	24.87 (22.59–27.63)
Waist circumference, cm	77 (71–83.5) (n = 992)	78 (71–84) (n = 133)	76 (71–83) (n = 859)
Hip circumference, cm	100 (95–106) (n = 991)	100 (95–106) (n = 133)	100 (95–106) (n = 858)
Current or former smoker, %	46.2	41.5	46.9
Physical activity score†	7 (6–8) (n = 991)	7 (6–8) (n = 134)	7 (6–8) (n = 857)

* Except where indicated otherwise, values are the median (interquartile range [IQR]). Selection bias (subjects used in this analysis versus subjects not used) was determined using the independent 2-tailed *t*-test for normally distributed data with the mean ± SD, the Wilcoxon rank sum test for non-normally distributed data with the median and IQR, and the chi-square test for categorical data expressed in percentages. There were no significant differences between groups. BMI = body mass index.

† From 3 (bad) to 12 (good).

**Table 2:** Morphologic predictors of THA*

	Control group without THA (n = 243 hips)	Case group with THA (n = 25 hips)	*P*†
Characteristic			
Age, years	55 (49–59)	58 (54–61)	0.012
Height, mean ± SD meters	1.61 ± 0.057	1.64 ± 0.05	0.015
Weight, kg	65.5 (58.7–71.8)	68 (61–80.2)	0.078
Pelvis			
Distance between center of femoral heads, mm	226.04 (217.97–232.56)	230.69 (225.63–240.97)	0.004
Interacetabular width, mm	153.52 (146.98–158.85)	157.88 (154.20–162.88)	0.011
Acetabulum			
Acetabular width, mm	61.3 (59.23–63.86)	62.45 (59.39–64.38)	0.401
Acetabular depth, mean ± SD mm	23.47 ± 3.53	23.63 ± 3.32	0.832
Acetabular depth-to-width ratio, mean ± SD	0.381 ± 0.053	0.380 ± 0.402	0.937
Lateral center edge angle, mean ± SD degrees	34.32 ± 6.77	29.54 ± 7.68	0.001
Extrusion index (ratio)	0.185 (12–25)	0.25 (17–34)	0.009
Acetabular index, degrees	4.125 (2.4–6.39)	5.32 (2.74–10.83)	0.013
Sharpe's angle, degrees	37.24 (34.62–39.53)	38.37 (35.58–39.68)	0.490
Acetabular tilt, degrees	55.25 (51.65–57.72)	56.08 (52.23–59.59)	0.218
Proximal femur			
Femoral head diameter, mm	54.99 (52.93–57.25)	55.54 (53.34–58.35)	0.217
Modified triangular index height, mean ± SD mm	26.92 ± 2.22	28.53 ± 2.94	0.001
Alpha angle, degrees	45.75 (43.29–53.95)	62.35 (46.52–83.6)	0.001
Minimum femoral neck width, mm	301.12 (287.86–317.94)	324.76 (310.61–381.25)	<0.0005
Femoral neck length, mm	54.47 (49.50–57.68)	56.02 (52.47–59.92)	0.195
Femoral neck length-to-width ratio	0.179 (0.161–0.198)	0.168 (0.147–0.190)	0.051
Femoral head-to-neck ratio	0.183 (0.175–0.189)	0.172 (0.153–0.178)	<0.0005
Femoral neck shaft angle, degrees	129.88 (126.06–133.89)	130.17 (126.95–134.68)	0.375
Modified proximal femoral angle, degrees	82.79 (78.53–85.88)	83.01 (80.75–86.97)	0.318
Minimum joint space width, mm	3.85 (3.67–4.33)	3.60 (2.99–4.03)	0.026
Dichotomous variables, %			
Protrusio acetabuli	0.82	0.00	0.822
Coxa profunda	52.67	48.99	0.192
Crossover sign (acetabular retroversion)	4.53	0.00	0.333
Triangular index	3.29	32.00	<0.0005

* Except where indicated otherwise, values are the median (interquartile range [IQR]). Of the 290 hips (145 subjects) studied, 22 were not included due to an exclusion criterion. The remaining 268 hips (135 subjects) comprised 25 hips of subjects who had undergone total hip arthroplasty (THA) and 243 hips of control subjects who had not.

† By independent 2-tailed *t*-test for normally distributed data, by Wilcoxon rank sum test for non-normally distributed data, and by Fisher's exact test for categorical data.

**Figure 2:**
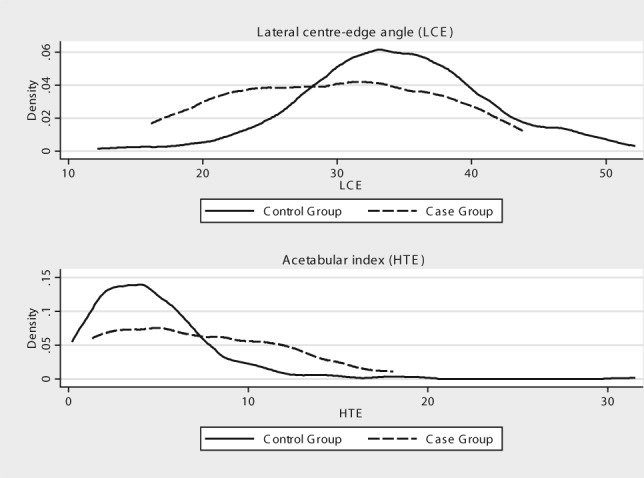
Kernel density plots of lateral center edge angle and acetabular index.

A number of morphologic variables were initially identified as being significantly associated with 19-year THA risk in univariate analyses (Table 2). These included a number of proximal femur measures; the alpha angle, triangular index (and its modification, the modified triangular index height), and femoral neck width were all greater in the patients, whereas the femoral head-to-neck ratio was greater in the controls. These results may be summarized as demonstrating a higher prevalence of cam deformity in the patients. In addition, a number of acetabular measures were significantly associated; the acetabular index and extrusion index were greater in patients, whereas the lateral center edge angle was smaller in patients. These results may be summarized as demonstrating a tendency toward acetabular dysplasia in the patients. A subgroup of the patients with THA could have had acetabular overcoverage (pincer deformity), which would be offset by the remaining patients having dysplasia and obscured by calculation of the mean or median values. However, the kernel density plots (Figure 2) did not demonstrate a clear bimodal distribution in the patients with THA.

Multivariate analysis using logistic regression (Table 3) revealed that the alpha angle, acetabular index, lateral center edge angle, and extrusion index remained significantly associated with THA after adjusting for age and BMI, whereas other morphologic variables lost statistical significance. These morphologic variables were then additionally adjusted for baseline radiographic OA evident at year 2 (osteophytes and joint space narrowing). There was little effect on the association of the alpha angle, lateral center edge angle, or extrusion index; however, the acetabular index became nonsignificant after adjusting for the presence of osteophytes. This indicates that the alpha angle, lateral center edge angle, and extrusion index were independently associated with the development of end-stage OA requiring THA by year 20, regardless of epidemiologic variables or preexisting OA at year 2.

**Table 3:** Logistic regression analysis*

Morphologic parameter	No adjustment, OR (*P*)	Adjusted by height, OR (*P*)	Adjusted by BMI, OR (*P*)	Adjusted by BMI and age, OR (*P*)	Adjusted by age and baseline JSN, OR (*P*)	Adjusted by age and baseline osteophytes, OR (*P*)
Extrusion index (ratio)	1.056 (0.002)	1.056 (0.002)	1.056 (0.002)	1.061 (0.001)	1.063 (0.007)	1.064 (0.005)
Acetabular index, degrees	1.131 (0.021)	1.144 (0.052)	1.137 (0.013)	1.161 (<0.0005)	1.151 (0.007)	1.121 (0.093)
Modified triangular index height, mm	1.306 (0.011)	1.242 (0.083)	1.302 (0.011)	1.291 (0.011)	1.296 (0.008)	1.195 (0.114)
Alpha angle, degrees	1.056 (<0.0005)	1.052 (0.001)	1.056 (<0.0005)	1.057 (<0.0005)	1.052 (0.001)	1.0452 (0.006)
Lateral center edge angle, degrees	0.906 (0.004)	0.906 (0.004)	0.903 (0.003)	0.887 (0.002)	0.880 (0.001)	0.891 (0.004)

* Odds ratios (ORs) were calculated using total hip arthroplasty by year 20 (outcome variable) and morphologic parameters at year 2 (exposure variable) (n = 268 hips). BMI = body mass index; JSN = joint space narrowing.

In order to assess whether these morphologic variables were independent of each other in predicting the requirement for THA, alpha angle and lateral center edge angle were added in to the model. The addition of further variables would have resulted in overfitting of the model and loss of statistical validity. Spearman correlation indicated a close association of extrusion index and lateral center edge angle (r = −0.77, *P* < 0.001), as would be expected since they both measure acetabular coverage. Therefore, extrusion index was dropped from the model. Based on 268 hips, for the alpha angle as an independent predictor of THA, the increase in risk of THA per 1° increase in alpha angle was 5.8% (95% confidence interval [95% CI] 2.3–9.3) (*P* < 0.001). For the lateral center edge angle as an independent predictor of THA, the increase in risk of THA per 1° reduction in lateral center edge angle was 10.5% (95% CI 2.0–18.2) (*P* = 0.017).

Table 4 demonstrates that the levels of agreement were good for the key morphologic variables identified from Table 2 and the subsequent regression analysis. Thus, the reliability of measurements was confirmed.

**Table 4:** Interobserver reproducibility and intraobserver repeatability

Parameter	Coefficient*	Interuser agreement (left hip, right hip)	Intrauser agreement (left hip, right hip)
Lateral center edge angle	ICC†	0.86, 0.63	0.95, 0.97
Extrusion index	ICC†	0.89, 0.80	0.95, 0.98
Acetabular index	ICC†	0.74, 0.61	0.96, 0.94
Triangular index	Kappa statistic‡	0.74, 0.74	0.74, 0.78
Alpha angle	ICC†	0.64, 0.52	0.77, 0.85

* ICC = intraclass correlation coefficient.

† Interuser agreement was based on 10 subjects, 3 raters; intrauser agreement was based on 10 subjects, 1 rater (3 repeated measures).

‡ Interuser agreement was based on 10 subjects, 2 raters; intrauser agreement was based on 10 subjects, 1 rater (2 repeated measures).

## DISCUSSION

This unique population-based study demonstrates that measures of hip joint morphology are associated with THA for end-stage OA over a followup period of 19 years in middle-aged and elderly women. These measures predict THA independent of evidence of baseline radiographic OA and, more important, independent of each other. This suggests that it may be worth assessing these simple-to-measure variables on standard radiographs to assess a woman's risk of later requirement for THA.

Cam deformities may be assessed on an AP pelvis radiograph by the alpha angle (18, 33) or triangular index (16), which measures asphericity of the head, or by the head-to-neck ratio, which indicates offset at the head–neck junction (19). Because cam deformities involve the anterolateral aspect of the head–neck junction, the AP pelvis radiograph is not necessarily the optimum projection by which to visualize abnormality (33). The triangular index was introduced as an alternative to measurement of the alpha angle from an AP pelvis radiograph and is less sensitive to varying femoral rotation, although both are highly interrelated (16). The results from the present study demonstrate that the alpha angle measured on an AP pelvis radiograph has a significant independent association with future risk of THA and is thus a useful clinical tool that can be used in assessing risk of THA for end-stage OA.

Historically, cam deformities have been termed “pistol-grip” (8) and “head-tilt” (7). Previous reports of the importance of a cam deformity in relation to OA are limited in their quality. The majority of studies are based on selected cohorts of patients with known childhood hip disease, OA, or hip pain and usually do not have radiographs of the subjects involved before the development of symptoms or OA (1, 7, 8, 12, 34, 35). Two large population-based studies, by Gosvig et al (13) and Doherty et al (36), have clearly associated the presence of cam deformity with OA. However, because of their cross-sectional nature, both studies are limited compared with ours and its 19-year followup. Furthermore, the outcome measure for OA in the study by Gosvig et al (13) was joint space narrowing. In the context of the projected increase in burden of THA (37), we consider THA to be a more clinically important surrogate marker of end-stage OA. Lynch et al (38) conducted a nested case–control study in a longitudinal cohort of elderly women (the Study of Osteoporotic Fractures) and examined associations of incident hip OA with variations in proximal morphology, assessed by active shape modeling. They found no association of incident OA and “pistol-grip”–shaped hips. It should be noted that this cohort was rather older than ours at baseline (mean age 71 years), and the followup period was shorter (8 years). Interestingly, from an analysis of the same cohort, Javaid et al (39) noted that increased femoral neck width was associated with prevalent and incident OA. This finding is consistent with our data.

Acetabular dysplasia has long been recognized as a risk factor for OA. The lateral center edge angle was first described by Wiberg (6) in 1939 as a quantitative measure of dysplasia and was found to be associated with an increased risk of OA when it was <20°. However, the evidence from subsequent case–control (40–42) and cross-sectional population-based (13, 43–45) studies is variable. Longitudinal studies in selected cohorts have also found conflicting evidence to support the use of parameters such as the center-edge angle, acetabular index, and extrusion index (10, 46). One population-based study with 8-year followup found that the center-edge angle was independently associated with the development of OA (11). Those data are in keeping with those of our study.

Acetabular overcoverage (pincer deformity [23]) has been linked to OA (14) and may also be described by the same parameters as those of dysplasia. The present study demonstrated that lateral center edge angle had a significant association with future risk of THA and that these were inversely related, meaning that in this population, it was subclinical dysplasia rather than pincer deformity that predicted THA. It is possible that a small proportion of the patients with THA developed end-stage OA secondary to a pincer deformity. Such cases of pincer deformity might have been obscured by a majority of cases of dysplasia; however, the kernel density plots did not show a bimodal distribution to support this contention. Nevertheless, it is plausible that such a relationship may become apparent in larger cohorts.

An abundance of reports link radiographic OA grade to risk of future THA (5). The purpose of measuring baseline (year 2) radiographic OA in the present study was so that effect size of morphologic parameters could be adjusted for baseline prevalence of OA in order to assess the independent effect of morphology on risk of THA. This is important, because in early OA secondary osteophyte formation at the femoral head–neck junction may be misinterpreted as a cam deformity. In late OA, femoral head collapse and osteophytes may produce a “pistol-grip” appearance (47). Therefore, it is important to account for this potential source of error in the analysis. As demonstrated earlier, there was a higher baseline prevalence of radiographic OA in the case group than in the control group, which undoubtedly contributed to those individuals in the case group undergoing THA during the 19-year followup period. However, the effect size of the morphologic variables remained significant even after the baseline OA was taken into account.

Ideally, our baseline assessment of morphology in this cohort would have occurred at the onset of skeletal maturity. This would clarify the association of the cam deformity and OA, and potentially confirm a causal relationship. No studies have tracked cases from the onset of skeletal maturity. In a sibling study of femoroacetabular impingement, Pollard et al (32) noted that many siblings of patients treated for cam impingement also had cam deformities themselves, in the absence of degenerative change. This observation, together with the findings in the current study, suggests that a cam deformity may predate alterations in morphology due to OA. We are not aware of any other study that has demonstrated this contribution of morphologic variables, independent of radiographic OA, to the risk of THA. Furthermore, no previous data indicate that these significant parameters are independent of each other. This study has clearly demonstrated that the alpha angle and lateral center edge angle represent measurements that are important in predicting THA risk individually.

This study has a number of potential limitations. Although AP pelvis radiographs have obvious limitations compared with 3-dimensional modalities when assessing joint morphology, they are inexpensive, easily accessible, and available for hundreds of thousands of patients worldwide, often obtained for other reasons. In addition, pelvis radiographs are available in many other large cohort study populations. The authors recognize that THA is a surrogate measure of end-stage OA in the hip. Other measures of OA include clinical severity scoring and radiographic grading, both of which may also be argued to be surrogate measures of disease state. THA is a surgical end point that has been previously validated as an OA outcome measure (48) and is highly relevant to both patient and practitioner as it indicates progression to end-stage disease. It should be noted that the UK has a taxpayer-funded public health system, and thus, all individuals in the present study may be considered to have equal access to THA with no personal expense. A limitation of this study was that our cohort consisted of only women, and therefore, the results may not be applicable to men. The incidence of a cam deformity in the asymptomatic male population approaches 20% (32), and it is currently not known why only a proportion of these individuals develop pain or OA. These findings therefore need to be reproduced in other populations.

In conclusion, a new software package called Hip Morf 2.0 was developed and validated. It measures 23 morphologic parameters around the hip joint. Hip Morf 2.0 was used to investigate the relationship between variations in hip morphology and 19-year risk of THA for end-stage OA in the hip in a group of 145 women. THA was used as the only outcome measure to assess whether an individual had developed end-stage OA. The present study found that the presence of a cam deformity, or acetabular dysplasia, as assessed by specific parameters, was significantly associated with future risk of THA, even after those parameters were adjusted for baseline height, age, BMI, and radiographic OA. An increase in alpha angle of 1° independently equated to a 5.8% increase in the 19-year risk of THA. Similarly, each 1° reduction in lateral center edge angle equated to a 10.5% increase in the 19-year risk of THA. Each of these morphologic parameters may be important predictors that can be simply used in an outpatient setting to assist in stratifying risk of future THA. These findings justify further investment in research investigating the role of early surgery that addresses such deformities and its efficacy in reducing the requirement for THA.
